# Two Surgeons Are Faster than One: A Comparative Study to Evaluate Efficiency and Outcomes in Reduction Mammaplasty

**DOI:** 10.1007/s00266-026-05999-6

**Published:** 2026-06-05

**Authors:** Iselin Saltvig, Salvatore Giordano

**Affiliations:** 1 Department of Plastic and General Surgery, Turku University Hospital, and the University of Turku, Turku, Finland; 2 Department of Surgery, Satasairaala Hospital, Pori, Finland

**Keywords:** Reduction mammoplasty, Breast reduction, Co-surgeon, Co-surgery, Quality improvement, Plastic surgery

## Abstract

**Background:**

The increasing demand for reduction mammaplasty has heightened the need for cost-effective and efficient surgical practices. Evidence from other surgical fields suggests that co-surgeon teams can improve operative efficiency.

**Aims and Objectives:**

This study aimed to compare operative time and outcomes in reduction mammaplasty performed by co-surgeons (CS) versus a single surgeon (SS).

**Materials and Methods:**

We conducted a retrospective cohort study including all consecutive patients who underwent primary reduction mammaplasty in a 20-years period. All procedures were led by attending faculty surgeons, with assistants being either residents or other attendings.

The primary outcome was operative time; secondary outcomes included intraoperative blood loss; resection weight, postoperative complications, reoperations, and length of hospital stay.

**Results:**

A total of 1716 patients were included: 855 (49.8%) underwent surgery with a SS, and 861 (50.2%) with a CS. Demographic and comorbidity profiles were mostly similar across the groups. Operative time was significantly shorter in the CS group (119.6 vs. 136.1 minutes, *p* < 0.001). Postoperative complication rates and early reoperations were similar between groups. However, CS cases had a longer mean hospital stay (0.6 vs. 0.4 days, *p* < 0.001). Late reoperations (>30 days post-op), particularly for dog-ear corrections were more common in the CS group (11.7 vs. 3.0%, *p* < 0.001). Multivariate analysis showed that higher BMI and smoking were significant predictors of complication occurrence.

**Conclusion:**

Using co-surgeons in reduction mammaplasty significantly reduces operative time. However, it may be associated with longer hospital stay and increased long-term reoperation rates. Further studies are needed to evaluate the cost-benefit ratio of this approach.

**Level of Evidence III:**

This journal requires that authors assign a level of evidence to each article. For a full description of these Evidence-Based Medicine ratings, please refer to the Table of Contents or the online Instructions to Authors www.springer.com/00266.

## Background

Breast hypertrophy (BH) can cause significant physical and psychological distress, including back, neck, and shoulder pain, headaches, dermatological issues, difficulty finding suitable clothing, and reduced self-esteem. Reduction mammaplasty has been shown to alleviate these symptoms and improve patients’ overall quality of life [1, 2]. First described in the late 1800s [3], breast reduction is now a routine and generally safe surgical procedure [4]. However, complications do occur, potentially compromising outcomes, increasing healthcare costs, and negatively impacting patient well-being.

As demand for reduction mammaplasty continues to grow optimizing surgical efficiency and outcomes is increasingly critical. According to the American Society of Plastic Surgeons, breast reduction procedures increased by 54% since 2019 [5]. Common strategies for improving outcomes include careful patient selection, preoperative optimization, and tailoring surgical techniques. Known risk factors for complications include elevated body mass index (BMI), diabetes mellitus (DM), cardiovascular disease (CVD), smoking [6], larger breast volume, and increased sternal notch-to-nipple distance [7]. Additionally, prolonged operative time has been linked to increased risk of complications such as bleeding, hypothermia, hypotension, and surgical site infections (SSI) [8–11].

Evidence from other surgical specialties suggests that involving a co-surgeon (CS) may significantly reduce operative time without increasing complication rates [12–17]. The co-surgeon model has also been associated with enhanced surgical training opportunities [14, 18–21] and reduced surgeon burnout [21].

Despite these potential benefits, the impact of co-surgeons in reduction mammaplasty (RM) remains largely unexplored. Investigating this model could reveal opportunities to improve surgical efficiency, support resident training, and increase case throughput without compromising patient safety.

In this study, we aimed to compare operative time and short-term outcomes of RM performed by co-surgeons (CS) versus a single surgeon (SS). We hypothesized that the use of co-surgeons would result in shorter operative times without an increase in complication rates, potentially offering both clinical and economic advantages.

## Methods

We conducted a retrospective cohort analysis of patients with BH who underwent breast reduction procedures by review of medical journals, including all consecutive patients who underwent primary reduction mammoplasty (RM) from January 2005 until December 2024 at a single academic institution. The study was conducted in accordance with the ethical principles of the World Medical Association Declaration of Helsinki and was approved by the local institutional review board (T145/2017). Owing to the retrospective nature of the study and anonymization of patient data, the requirement for individual informed consent was waived.

Comprehensive demographic and clinical data were extracted from electronic medical records, including preoperative assessments, and postoperative follow-up data from outpatient visits.

The number of surgeons assigned to each procedure was determined by the department’s booking operational manager. However, there was a departmental convention of scheduling two surgeons (co-surgeons, CS) for the last surgical case of each day.

### Inclusion and Exclusion Criteria

Inclusion criteria were limited to consecutive female patients older than 18 years who underwent bilateral reduction mammaplasty with a minimum follow-up period of 3 months and no previous breast reduction procedures.

Patients younger than 18 years, male gender, or who underwent unilateral breast reductions, mastopexies, and procedures involving oncological resections were excluded.

National recommended criteria for RM eligibility during the study period included symptoms of cervical syndrome refractory to physiotherapy, a jugulum–nipple distance >27 cm, and a body mass index (BMI) of ≤ 30 kg/m^2^ (≤32 kg/m^2^ before 2015).

Patients were categorized into two groups based on the number of surgeons: single surgeon (SS) or co-surgeons (CS).

### Surgical Technique and Surgeon Roles

Most procedures were performed using Wise pattern markings and a superomedial pedicle. In all cases, surgery was led by a faculty (specialist) surgeon. Assistants included either attending surgeons or residents with at least three years of surgical training.

For the purposes of this study, the co-surgeon (CS) group was defined as procedures performed by two surgeons (one attending-level surgeon together with either another attending surgeon or an experienced resident) working concurrently. In this setting, both surgeons actively participated in the operation, typically performing different components simultaneously (e.g., one surgeon performing tissue resection on one breast while the other performed closure on the contralateral side). Residents, when present, functioned as co-surgeons.

The single-surgeon (SS) group comprised procedures performed by one attending surgeon assisted by nursing staff only, without the involvement of a second surgeon.

### Outcomes

The primary outcome measure was operative time. Secondary endpoints included postoperative complication rates, specific wound-healing complications, and late complications. These included hematoma, seroma, wound dehiscence, fat necrosis, cellulitis, abscess, and systemic medical complications.

Hematomas and seromas were defined as subcutaneous accumulations of blood or serous fluid, respectively, that required percutaneous aspiration or surgical evacuation. Intraoperative blood loss was measured by the volume collected in suction canisters, including fluid obtained from manually expressed surgical swabs.

Wound dehiscence was defined as a full-thickness separation of wound edges extending more than 2 cm, with or without infection. Skin necrosis was characterized by a clearly demarcated necrotic area exceeding 1 cm in width. Fat necrosis was defined as a firm, palpable mass measuring ≥1 cm in diameter that persisted for more than three months postoperatively.

Surgical site infections included localized cellulitis or abscesses requiring oral or intravenous antibiotic therapy, with or without surgical intervention. Nipple necrosis was defined as partial or complete ischemia or necrosis of the nipple-areolar complex. Infections were recorded based on the initiation of antibiotic treatment. In Finland, all prescribed medications are logged in a centralized national database, allowing detection of cases even when patients did not directly contact the surgical department.

Anaesthetic or systemic medical complications included deep vein thrombosis, pulmonary embolism, and other perioperative adverse events.

Postoperative follow-up evaluations were performed by plastic surgeons, typically at 1, 3 and 6–9 months postoperatively.

### Statistical Analyses

Continuous variables were summarized as means with standard deviations (SD), while categorical variables were presented as frequencies and percentages. The normality of continuous data distributions was assessed using histograms, skewness, kurtosis, and the Kolmogorov–Smirnov test. Comparisons between the co-surgeon (CS) and single-surgeon (SS) groups were performed using Student’s t-test for normally distributed continuous variables. Categorical variables were compared using Pearson’s Chi-square test or Fisher’s exact test, as appropriate. Multivariable logistic regression was used to assess independent risk factors for complications based on whether surgeries were performed by one or two surgeons, with adjusted odds ratios provided. The Hosmer–Lemeshow test was used to assess the goodness-of-fit of the logistic regression model; the resulting *p*-value was 0.646, indicating a good fit. All tests were two-sided, and a *p*-value ≤ 0.05 was considered statistically significant. All statistical analyses were conducted using IBM SPSS Statistics, version 30.0 (Armonk, NY, USA).

## Results

A total of 1716 patients were included in this study, with 855 (49.8%) undergoing reduction mammaplasty (RM) with a single surgeon (SS) and 861 (50.2%) with co-surgeons (CS). Patient demographics are summarized in Table 1. The mean age was similar between the groups (43.8 years for SS vs. 45.0 years for CS; *p* = 0.062). Mean BMI was also higher in the CS group (27.0 for SS vs. 27.5 for CS; *p* < 0.001). Most of comorbidities were similar among the groups. However, patients in the CS group had a significantly higher rate of hypertension and smokers (Table 1). The mean follow-up duration was significantly longer in the CS group (9.5 months vs. 5.1 months; *p* < 0.001).

**Table 1:** Demographics of patients at time of study

	One attending surgeon (n = 855)	Two attending surgeons (n = 861)	*p*-value
Age (mean ± SD)	43.8 ± 15.0	45.0 ± 12.5	0.062
Mean BMI (kg/m^2^)	27.0 ± 2.5	27.5 ± 3.2	<0.001
Any comorbidity	399 (46.7%)	431 (50.1%)	0.160
Diabetics	21 (2.5%)	28 (3.3%)	0.322
Hypertension	109 (12.7%)	153 (17.8%)	0.004
Pulmonary disease	62 (7.3%)	77 (8.9%)	0.199
Depression	106 (12.4%)	131 (15.2%)	0.091
Smokers	106 (12.4%)	137 (15.9%)	0.037
Herbal supplement	61 (7.1%)	53 (6.1%)	0.853
Follow-up (months)	5.1 ± 11.9	9.5±14.5	<0.001

The mean operative time was significantly shorter in the CS group compared to the SS group (121.6 vs. 142.3 minutes, *p* < 0.001, Table 2). The reduction in operative time observed in the CS group likely reflects parallel operative workflow between two surgeons.

**Table 2:** Comparison of peri-operative parameters in the two groups of patients

	One attending surgeon (n = 855)	Two attending surgeons (n = 861)	*p*-value
Operative time (min, mean ± SD)	136.1±35.7	119.7±29.7	<0.001
Resection weight from right breast (g, mean ± SD)	594.8±258.9	641.8±300.4	<0.001
Resection weight from left breast (g, mean ± SD)	604.3±265.0	647.4±296.8	0.002
Blood loss (ml, mean ± SD)	259.0±183.8	310.3±188.4	<0.001
Hospital stay (days, mean ± SD)	0.45±0.67	0.63±0.68	<0.001

Resection weight and intraoperative blood loss were significantly higher in the CS group. Similarly, the mean duration of hospitalization was significantly longer in the CS group (0.63 days vs. 0.45 days for SS; *p* < 0.001, Table 2).

Complication rates were similar with 11.2% in the SS group and 13.1% in the CS group (*p* = 0.230, Table 3). While most postoperative complications were comparable between groups, the CS group had a significantly longer hospital stay (0.6 vs. 0.2 days; *p* < 0.001). Additionally, the rate of reoperations occurring more than 30 days after the primary surgery was significantly higher in the CS group (11.7 vs. 3.0%; *p* < 0.001). Most of late reoperations were due to dog-ear correction and occurred more often in CS group (Table 3). A multivariable logistic regression analysis revealed that higher BMI and smoking were significant predictors of complication occurrence (Table 4).

**Table 3:** Postoperative complications at follow-up

	One attending surgeon (n = 855)	Two attending surgeons (n = 861)	*p*-value
Patients with complications	96 (11.2%)	113 (13.1%)	0.230
*Complications*			
Superficial wound infection (received antibiotics <30 days)	26 (3.0%)	35 (4.1%)	0.252
Deep wound infection (revision in local anaesthetics)	46 (5.4%)	45 (5.2%)	0.887
Wound dehiscence (T-junction mostly, need for revision -local/general)	14 (1.6%)	24 (2.8%)	0.106
Fat necrosis (need for operation)	10 (1.2%)	16 (1.9%)	0.243
Hematoma (need for operation)	25 (2.9%)	29 (3.4%)	0.598
Nipple necrosis (partial/total, requiring extra follow-up/procedure)	18 (2.1%)	27 (3.1%)	0.180
Reoperation <30 days	32 (3.7%)	27 (3.1%)	0.490
Reoperation at follow up, more than 30 days post operatively	30 (3.0%)	101 (11.7%)	<0.001
Reoperation for dog-ear	26 (5.8%)	100 (7.9%)	0.145

**Table 4:** Multivariable logistic regression was used to assess independent risk factors for complications based on whether surgeries were performed by one or two surgeons, with adjusted odds ratios provided

	Odd ratios	95% Confidence interval	*p*-value
BMI	1.12	1.04-1.20	0.004
Smoking	1.89	1.20-2.96	0.006
Operative time	1.01	1.00-1.01	0.051
Resection weight	1.00	1.00-1.00	0.061
Depression	1.37	0.79-2.38	0.257
Pulmonary disease	1.39	0.72-2.65	0.324
Blood loss	1.00	0.99-1.00	0.647
Hypertension	0.88	0.49-1.58	0.682
Co-surgeon	1.10	0.73-1.60	0.688
Diabetes	0.85	0.28-2.58	0.775

## Discussion

This retrospective cohort study evaluated the impact of single-surgeon (SS) versus co-surgeon (CS) teams on operative efficiency and outcomes in reduction mammaplasty (RM). Our findings demonstrate that employing a CS significantly reduces operative time by an average of 17 minutes without increasing short-term complication rates, reinforcing evidence from other surgical disciplines [12–17, 22–29]. Importantly, this efficiency gain is not simply a result of a second surgeon performing the contralateral procedure independently. In our workflow, the co-surgeon overlaps with tissue reduction on one side while assisting and/or performing closure on the contralateral side, enabling parallel task execution and more streamlined intraoperative decision-making. These results highlight the practical value of the co-surgeon model in optimizing operative efficiency and support its broader application in both RM and other surgical procedures [12, 20].

Interestingly, despite the shorter operative time, the CS group had significantly longer hospital stays (0.63 vs. 0.45 days, *p* < 0.001). This is contrary to reports from microsurgical breast reconstruction studies [23, 25, 28], where CS use often shortened hospital stays. In our context, the longer stay likely reflects non-clinical factors such as scheduling practices where CS cases were often performed last in the day and kept overnight for routine observation rather than clinical necessity. Perioperative characteristics and complication rates between groups were otherwise similar, suggesting hospitalization duration was not medically driven.

Although complication rates did not differ significantly between groups, late reoperations (>30 days postoperatively) were more frequent in the CS group (11.7 vs. 3.0%, *p* < 0.001). These were mostly minor revisions such as dog-ear corrections, rather than complications from asymmetry, which literature suggests is a common cause of revision in RM [17, 30]. The increased revision rate in the CS group may reflect heightened attention to aesthetic refinement or more thorough intraoperative assessments made possible by the presence of a co-surgeon. These findings are somehow contradictory with previous research, where the CS model may facilitate improved intraoperative decision-making and better long-term outcomes, supporting prior evidence of lower complication rates with dual-surgeon teams [15, 27, 28, 31–33]. The increased revision rates for the SS are in line with literature describing a trend of lowered over all complication rates when employing a co-surgeon approach [15, 27, 28, 31–33]. As preoperative demographics were similar, we do not believe the increased revision rate was due to selection bias, with more complicated cases being allocated to a single surgeon. Physiological variations, pre- and perioperative asymmetry are both described as challenges in bilateral procedures [17, 34], making RM more prone to need for revision due to inherent asymmetry. Interestingly, knowledge of our unit enabled us to determine that the late complications were largely revisions due to corrections of dog ears, in CS group, and not asymmetry.

Our study also raises important considerations for surgical education. In academic centers, the CS team often includes residents. While the almost 20-minute time savings may seem minimal in a relatively short procedure, it could represent a meaningful opportunity for teaching, as well as enhanced intraoperative communication. Furthermore, co-surgery could help mitigate surgeon fatigue and burnout, which are a growing concern in surgical disciplines, by distributing the workload [21]. An important consideration in interpreting these findings is the definition of the co-surgeon model used in this study. In contrast to some interpretations in the literature, co-surgery in our cohort did not reflect independent bilateral resections performed simultaneously by two primary surgeons. Rather, it involved two surgeons working in parallel on different components of the same procedure (e.g., resection on one side and closure on the contralateral side). As such, the observed reduction in operative time in the co-surgeon group is best understood as a function of parallel workflow and task-sharing rather than a direct comparison of two fully independent primary resections performed simultaneously.

From an economic perspective, reduced operative time may increase operating room throughput and overall surgical capacity. In our institution, operating room time is estimated at approximately €2700 per hour. A 20-minute reduction per procedure could translate to cost savings of around €900 per case. While we did not perform a formal cost analysis, literature suggests that the CS model is cost-effective in various surgical contexts [16, 25, 28, 32, 33]. Particularly in high-volume centers, these savings could scale substantially, potentially reducing waiting lists and improving access to RM.

To the best of our knowledge this is the first study exploring the impact of CS on RM, although previous study explored this topic in plastic surgery, and microsurgery. One of this study’s strengths lies in the large, single-institution cohort of 1716 patients with consistent and relatively long follow-up. Additionally, the use of Finland’s centralized prescription database enabled the capture of complications treated outside our department, improving the completeness of our data.

This study has several limitations. First, its retrospective design introduces the risk of selection and information bias. When encountering significant variations in our analysis, we attempted to investigate these when identified by chart reviews, as a measure to alleviate this limitation. However, there is a possibility that some data were not captured in the study. Group allocation was not randomized, and the institutional scheduling practice may have influenced which patients received co-surgeon care. While the allocation of a SS or a CS was done at the discretion of the booking secretary, we cannot completely exclude the possibility that the more complex cases were preferentially assigned to CS-teams. Second, although patient demographics and comorbidities were generally balanced, the CS group had slightly healthier profiles, potentially confounding outcome comparisons. Third, operative techniques, surgeon experience, and intraoperative decisions were not standardized or uniformly recorded, which may impact the generalizability of the findings. Finally, follow-up duration differed between groups, potentially affecting the detection of late complications. Finally, our data are from a single institution and, and while comprehensive, may be influenced by local tendencies and fail to reflect larger system trends.

Despite these limitations, the large cohort and real-world clinical setting offer valuable insights into the evolving role of co-surgery in plastic surgery. We provide valuable insights into the potential benefits and trade-offs of a co-surgeon approach in RM. The findings suggest that while co-surgery can improve operative efficiency, attention must be paid to postoperative care and long-term outcomes.

Our findings are particularly relevant to institutions seeking to increase efficiency, where the addition of an extra surgeon safely reduces surgical duration thereby potentially increasing capacity. It also sheds light on the importance of proper scheduling to optimally utilize this reduction of surgical duration. Further it is relevant to institutions with educational functions, supporting the inclusion of residents as CS.

Future research should explore whether the benefits of CS extend to higher-risk populations, such as patients with elevated BMI or comorbidities. Additionally, prospective multicenter trials with standardized protocols would strengthen the evidence base. A cost-benefit analysis that includes not only operative time and resource use but also long-term outcomes and patient-reported satisfaction (e.g., via BREAST-Q [35]) could further clarify the utility of co-surgery. Educational studies tracking the roles and training levels of both SS and CS team members would also help elucidate how co-surgery impacts resident learning and procedural outcomes.

## Conclusion

The use of co-surgeons in reduction mammaplasty significantly reduces operative time and appears to be a safe and effective strategy in the short term. However, the observed increase in hospital stays and late reoperation rates in the co-surgeon group highlights the need for further evaluation. While co-surgery may enhance surgical efficiency and provide educational opportunities, its long-term implications on patient outcomes and healthcare utilization should be carefully considered. Future prospective and randomized studies are warranted to better understand the balance between efficiency, safety, and cost-effectiveness in co-surgeon models.
